# Nervous Exhaustion

**Published:** 1876-11

**Authors:** 


### ARTICLE VIII.

### *Nervous Exhaustion.*

The word "exhaustion" is very significant, and very easily understood. It is derived from two words, *ex* out, and *haurire*, to draw. Put together, of course we have exhaustion, which means to draw out, to drain off, till nothing is left. We exhaust the water in a well, for instance, by drawing out, by pumping it dry. We exhaust our forests by cutting them down. We exhaust our resources by extravagant living. We become physically exhausted by great labor, strain, starvation, etc. Physical exhaustion has reference to the whole body, including the nerves. The powers of the stomach may be exhausted, causing inability to digest. The power of the muscles may be exhausted, causing physical weakness, inability to walk, to lift any weight, to perform any physical labor. The muscles may be exhausted in one or more ways; they may be exhausted by excessive labor, by inability of the stomach to digest food, by fevers and various diseases, by great strain, by inability of the brain to supply nerve force. The brain and nerves may be exhausted in the same way. Exhaustion may be temporary or permanent; a day's labor may cause temporary exhaustion. Temporary exhaustion comes from the temporary using up of nervous tissue and nervous supply, and a night of sleep, with appropriate food, restores it; or it may be a month of rest and recreation is needed. Permanent exhaustion comes from the destroying of the source of nervous supply, or the permanent injury of the nerves themselves. This will be made clear by a comparison of the nervous system to the old fashioned saw mill, located on a small stream of water on the border of a wood. A dam across the stream, uniting the opposite banks, stops the water in its course, and it backs up and fills the banks till the water overflows. Now the accumulated water is turned through a sluice unto the the wheel and the mill is set in operation and does the work of hundreds of men. An insufficient rain, or a a failure of the swamps and springs exhausts the water supply, and the mill stops till the water accumulates to fill the dam again, when the mill moves on. If the supply is only temporarily cut off, the delay of work is temporary; if permanently cut off, the mill cannot be run with profit, and is torn down, or else a steam engine is put in and the motive force manufactured by fire and water, which is more under human control than the rainfall. The nervous system is very much like a machine. It must have its daily and hourly supply of force in some way or other, or exhaustion is the result. We care more for our machinery, keep it bright and use it wisely, in order that it may last a long time. We use ourselves too often as if we were worth less than our horses, our dogs, or axes and saws, or tools that cost little and last long.

The victims of nervous exhaustion are numerous. They meet us at every turn. Their variety is numberless. There is the mother, exhausted with childbearing, and at the same time care of the household, by sleepless nights and deficient digestion and assimilation of food. There is the woman of fashion, whose beauty has faded and whose charms are gone, whom the demands of society have robbed of health, and who is a wreck mentally and physically. There is the hypochondriac, whose gloomy views of life, whose depression of spirts, whose mental debility, whose indisposition to activity, are a source of constant pain to himself and his friends. There is the melancholy one, whose dejected spirits and cast down manner, whose mental alienation and dismal condition lowers the happiness of all who come in contact with him. Then there is the victim of sexual excesses, whose nervous state often borders on, and runs into insanity, and whose state of nervous exhaustion causes more bodily suffering than almost any other nervous malady. We might add to these, nervous exhaustion from excessive care, anxiety, overstrain, business ventures that turn out badly. All these are easily recognized. There are also other milder forms, not causing hypochondria, melancholy, or much mental agony; but they make a great deal of trouble. We refer to those forms of nervous exhaustion which cause irritability, fretfulness, a disposition to criticise, find fault, scold, to get peevish, to be easily offended, to get excited on trivial occasions, to magnify trifles, to cry easily, to get angry and fly into a passion, to sleep poorly, to be dainty about food, to be unable to keep the mind on one thing, but run off unto something else, to be unable to keep a steady hand, or speak connectedly on any topic, even often to utter a sentence logically and coherently. These, and many more symptoms, are often the result of a weakened condition of the nervous system—in short, a mild form of nervous exhaustion.

Many do not recognize these forms of mental obliquity as diseases; but as moral defects, and treat them by censure. They should be recognized as physical diseases, and treated by hygiene. Children often receive unjust treatment from parents for peculiarities, when they should receive medical or hygienic treatment.

These nervous evils endanger the prosperity or mental character of the race. Their capability of being propagated is very great. The offspring of nervously exhausted parents are apt to possess the evil in an increased degree. Moderation, and a pleased, happy state, are the normal conditions of mind. Nervousness, indeed all nervous disorders, so to say, distort the harmony of life, hinder the haste which they generate, put an end to contemplation, and act in an unfriendly manner to moderation. Where nervousness dwells, prevails never bodily soundness of the individual; but liability to extremes of action and reaction. The person is now on the heights, now in the depths; now joyous, now so miserable and unhappy that he thinks he never had a joy in his life, and never expected one.

The causes of nervousness may be summed up in the following general heads:

1. They are inherited.
2. They arise from defective nutrition.
3. From overstrain.
4. From the use of stimulants.
5. From insufficient sleep.
6. From indulgence in vice and passion.
7. From scrofula.
8. From anything that deteriorates the physical constitution and lowers the character of the bodily candition.

These causes, singly or united, bring about a sort of nervous bankruptcy, of diseased blood, of dyspepsia, and with it innutrition. As has been said before, the normal activity of the nervous system is dependent on rich, healthy blood. Nervously exhausted people always have thin poor blood. It is deficient in fibrin and blood corpuscles. There is not force enough stored up in it to keep the wheels of life in a high state of activity, and so they go slowly, feebly, hardly at all.

In our age nervous exhaustion is in the ascendant. It crops out in every quarter. Our hothouse education generates it, by cultivating the mind at the expense of the body. Our sedentary ways of living generate it. Our haste after money, our risks, our anxieties, our cares, all help to bring on nervousness. Only the prudent and well organized escape; and even they sometimes are engulfed by the faults and treachery of others. It is high time for us to consider this matter in the light of science and common sense, and see if something cannot be done to relieve this generation from the curse of nervous exhaustion, and show people how to conduct their lives so that peace and serenity shall take the place of haste and excitement, and all its attending evils. In another chapter we shall point out the remedies.—*Editor of Herald of Health.*

